# Editorial: The role of gut microbiota in mediating the physiological and pathological effects of pollutant exposure in aquatic animals

**DOI:** 10.3389/fmicb.2026.1897934

**Published:** 2026-06-23

**Authors:** Yuhang Hong

**Affiliations:** 1 Key Laboratory of Application of Ecology and Environmental Protection in Plateau Wetland of Sichuan, Xichang University, Xichang, China; 2 Key Laboratory of Animal Disease Detection and Prevention in Panxi District, Xichang University, Xichang, China

**Keywords:** aquatic toxicology, environmental pollution, gut microbiota, host–microbe interactions, toxic mechanisms

Aquatic ecosystems are increasingly threatened by a broad spectrum of environmental pollutants, including pesticides, heavy metals, pharmaceuticals, antibiotics, microplastics, and engineered nanomaterials. These contaminants can enter rivers, lakes, reservoirs, estuaries, and aquaculture systems through agricultural runoff, industrial discharge, domestic wastewater, and plastic debris. In aquatic animals, pollutant exposure has been widely associated with oxidative stress, inflammation, metabolic disorders, developmental toxicity, immunosuppression, neurobehavioral alterations, and reduced ecological fitness. However, conventional aquatic toxicology has mainly focused on direct toxic effects on target organs such as the gill, liver, intestine, nervous system, and immune tissues. Increasing evidence now suggests that the gut microbiota should be considered an active biological interface between environmental exposure and host health.

The gut microbiota contributes to nutrient digestion, energy homeostasis, epithelial barrier maintenance, immune regulation, and gut–brain communication in fish and other aquatic animals (Butt and Volkoff, 2019; Egerton et al., 2018). Because aquatic animals continuously interact with surrounding water, sediment, feed, and suspended particles, their intestinal microbial communities are particularly sensitive to environmental fluctuations and contaminant exposure. Pollutant-induced dysbiosis may therefore not only serve as a biomarker of environmental stress but also participate in the progression of toxicity by altering microbial metabolites, intestinal permeability, inflammatory signaling, redox homeostasis, and host metabolic pathways.

Several lines of evidence support this emerging view. Microplastics can accumulate in the gastrointestinal tract and disturb intestinal homeostasis. In zebrafish, polystyrene microplastic exposure induced intestinal inflammation and oxidative stress and was accompanied by marked changes in both gut microbiota and metabolomic profiles (Qiao et al., 2019). Long-term dietary exposure to microplastics similarly triggered oxidative damage and pro-inflammatory responses in the gut of gilthead seabream, indicating that plastic particles may directly impair intestinal health in marine fish (Solomando et al., 2020). A recent meta-analysis further suggested that microplastics predominantly affect aquatic animal gut microbiota by reshaping community structure rather than simply changing richness or diversity, highlighting the need to move beyond alpha-diversity indices when interpreting microbiome toxicity (Shi et al., 2024). Pesticides and other emerging pollutants may also alter microbiota-linked host functions. A recent review focusing on freshwater animals emphasized that microplastics and pesticides can affect gut microbial communities and may contribute to behavioral and physiological impairments through microbiota-related pathways (Burgos-Aceves et al., 2024).

Heavy metals and antibiotics provide additional mechanistic evidence for microbiota-mediated toxicity. Cadmium exposure has been shown to disrupt the gut microbiota and aggravate neurotoxicity in zebrafish larvae through a V-ATPase-related mechanism, with germ-free zebrafish models supporting a causal role of microbiota in modulating Cd-induced neurotoxicity (Xu et al., 2023). Environmental antibiotics, even at sub-lethal and environmentally relevant concentrations, may induce dysbiosis and impair physiological homeostasis, development, and reproduction in fish and aquatic invertebrates, suggesting that microbiota disturbance is an overlooked component of ecological risk assessment for antibiotic pollution (Li et al., 2023). Together, these studies indicate that pollutant-induced toxicity cannot be fully understood without considering host–microbiota interactions.

This Research Topic, “*The Role of Gut Microbiota in Mediating the Physiological and Pathological Effects of Pollutant Exposure in Aquatic Animals*,” was established to advance this field by integrating aquatic toxicology, microbial ecology, host physiology, and omics-based approaches. The published articles cover diverse aquatic organisms and pollutant contexts, including triclocarban exposure in *Rana taihangensis* tadpoles, combined ZnO nanoparticle and glyphosate exposure in tadpoles, Ti_3_C_2_T_x_ MXene toxicity in *Daphnia magna*, mercury-associated microbiome variation in three-spined stickleback populations, oyster-associated bacteriome assembly in Yueqing Bay, and a review of mechanistic links between environmental pollutants, gut microbiota, and systemic disease (Zhao et al.; Han et al.; Xiang et al.; Kuizenga et al.; Lin et al.; Ma et al.). These contributions were published in Frontiers in Microbiology and collectively highlight gut microbiota, aquatic toxicology, environmental pollution, host–microbe interactions, and toxic mechanisms as the central keywords of the Research Topic.

The studies in this Research Topic illustrate three important advances. First, pollutant-induced gut dysbiosis is evident across multiple aquatic taxa, including amphibians, crustaceans, fish, and mollusk-associated microbial systems. Second, microbiota changes are increasingly being linked with host phenotypes such as development, growth, metabolism, mercury accumulation, and environmental adaptation rather than being treated as isolated community shifts. Third, the field is expanding from conventional pollutants to emerging materials such as MXenes and nanoparticles, reflecting the need to evaluate novel contaminants before their ecological impacts become widespread. Despite these advances, important challenges remain. Many studies still rely primarily on 16S rRNA sequencing and correlation-based interpretation, which limits causal inference. Future research should incorporate germ-free models, antibiotic-mediated microbiota depletion, fecal microbiota transplantation, probiotic supplementation, and microbial metabolite rescue experiments. Multi-omics strategies integrating metagenomics, metatranscriptomics, metabolomics, host transcriptomics, and immune or neuroendocrine endpoints will be essential for identifying functional mechanisms. More attention should also be paid to chronic low-dose exposure, pollutant mixtures, field validation, sex- and population-specific responses, and microbiota-based biomarkers for ecological risk assessment.

Overall, this Research Topic demonstrates that gut microbiota research is becoming a key frontier in aquatic toxicology. By moving from descriptive dysbiosis toward mechanistic and causal models, future studies will improve our understanding of how aquatic animals respond to pollutant stress and may provide new strategies for environmental monitoring, aquaculture health management, and pollution mitigation.
